# Using machine learning to determine age over 16 based on development of third molar and periodontal ligament of second molar

**DOI:** 10.1186/s12903-023-03284-5

**Published:** 2023-09-20

**Authors:** Shihui Shen, Zhuojun Zhou, Jian Wang, Linfeng Fan, Junli Han, Jiang Tao

**Affiliations:** 1 Department of General Dentistry, Shanghai Ninth People’s Hospital, Shanghai Jiao Tong University School of Medicine, Shanghai, China; 2 College of Stomatology, Shanghai Jiao Tong University, Shanghai, People’s Republic of China; 3 National Center for Stomatology, Shanghai, People’s Republic of China; 4 National Clinical Research Center for Oral Diseases, Shanghai, People’s Republic of China; 5 Shanghai Key Laboratory of Stomatology, Shanghai Research Institute of Stomatology, Research Unit of Oral and Maxillofacial Regenerative Medicine, Chinese Academy of Medical Sciences, Shanghai, People’s Republic of China; 6 Department of Radiology, Shanghai Ninth People’s Hospital, Shanghai Jiao Tong University School of Medicine, Shanghai, China

**Keywords:** Age determination, Juvenile criminal age, Machine learning, Second molar, Third molar, Periodontal ligament

## Abstract

**Background:**

Having a reliable and feasible method to estimate whether an individual has reached 16 years of age would greatly benefit forensic analysis. The study of age using dental information has matured recently. In addition, machine learning (ML) is gradually being applied for dental age estimation.

**Aim:**

The purpose of this study was to evaluate the development of the third molar using the Demirjian method (Demirjian_3M_), measure the development index of the third molar (I_3M_) using the method by Cameriere, and assess the periodontal ligament development of the second molar (PL_2M_). This study aimed to predict whether Chinese adolescents have reached the age of criminal responsibility (16 years) by combining the above measurements with ML techniques.

**Subjects & methods:**

A total of 665 Chinese adolescents aged between 12 and 20 years were recruited for this study. The development of the second and third molars was evaluated by taking orthopantomographs. ML algorithms, including random forests (RF), decision trees (DT), support vector machines (SVM), K-nearest neighbours (KNN), Bernoulli Naive Bayes (BNB), and logistic regression (LR), were used for training and testing to determine the dental age. This is the first study to combine ML with an evaluation of periodontal ligament and tooth development to predict whether individuals are over 16 years of age.

**Results and conclusions:**

The study showed that SVM had the highest Bayesian posterior probability at 0.917 and a Youden index of 0.752. This finding provides an important reference for forensic identification, and the combination of traditional methods and ML is expected to improve the accuracy of age determination for this population, which is of substantial significance for criminal litigation.

**Supplementary Information:**

The online version contains supplementary material available at 10.1186/s12903-023-03284-5.

## Introduction

A reliable and repeatable method for estimating age 16 would be highly valuable in forensics. Many countries and regions worldwide consider anyone over 16 criminally responsible for their actions, as does Chinese law (see Table 1). The International Federation of Gymnastics also requires female gymnasts to be at least 16 years old to participate in the Olympic Games.

**Table 1:** Legal content of People’s Republic of China (PRC) for the 16 years old

Law of PRC	Article	Legal content
Criminal Law	Article 17	If a person who has reached the age of 16 commits a crime, he shall bear criminal responsibility
Civil Code	Article 18	A minor aged 16 or above whose main source of support is the income from his own labor is deemed as a person with full capacity for performing civil juristic acts
Labour Law	Article 15	It is forbidden for employers to employ persons under the age of sixteen. Whenever a unit in culture and arts, sports and special arts and crafts needs to employ young persons under the age of sixteen, examination and approval procedures-shall be undertaken according to relevant regulations of the State and the employees thereof should be ensured the right of receiving compulsory education
Penalties for Administration of Public Security	Article 21	Under one of the following circumstances, the penalty of administrative detention shall not be executed against the person who has committed an act against the administration of public security, although such a penalty should be imposed on him/ her according to the provisions of this Law:(1) The person has attained to the age of 14 but not to the age of 16;(3) The person is over 70 years old; or (4) The person is pregnant or breast-feeds her own baby who is not one year old
Order of the State Council	Article 72	People who drive electric bicycles and motorized wheelchairs for the disabled must be at least 16 years old;

Extensive research has been conducted in recent decades on age estimation, and various methods have been suggested and applied to different age and ethnic groups [1–8]. Previous methods used for dental age estimation primarily relied on assessing the root development of the seven mandibular teeth. However, most of these 7 teeth fully develop before age 14, which makes it challenging to determine whether an individual has reached the age of 16. As a result, researchers have shifted their focus to the development of the third molars and the periodontal ligament.

The third molar can be a flexible, feasible, and reliable indicator of whether a person is 16 years of age since its developmental stage is longer than that of other teeth [9]. The development and mineralization of the third molar continue until after the age of 18. Therefore, an increasing number of studies focus on third molars for age estimation [10–14].

The original Demirjian staging system, primarily developed for assessing the dental age of adolescents based on the evaluation of seven mandibular teeth, may not be suitable for estimating the age of individuals aged 16 and above. The difference between chronological and dental age at this stage can exceed 1 year [15]. To address this issue, researchers began applying the Demirjian staging system to mandibular third molars (Demirjian_3M_). Cameriere proposed an approach that measures the index of the mandibular third molar (I_3M_) to infer whether the person is older or younger than 18, then applied it to 16-year-olds [16].

In addition to tooth root development, researchers have focused on the relationship between the development of the periodontal ligament and age. Olze A et al. divided periodontal ligament development into 4 stages [17]. Many subjects in the Chinese population have fused tooth roots or narrow bifurcations in their mandibular third molars, making it impossible to classify them into any stage. To overcome the limitations of the previous standards set by Olze et al., Guo et al. introduced a new classification based on the visibility of the periodontal membrane. The optimized criteria proposed by Guo et al. were adopted here since this study uses a sample from China [18]. Considering that the third molars may not have fully developed by the age of 16 in some individuals, the periodontal ligament of the second molar (PL_2M_) was utilized for the first time to assess whether children have reached the age of 16 or above.

Machine learning (ML) is gradually being applied to study dental and bone age [19–21]. As a type of artificial intelligence, ML can not only effectively estimate dental age but also has a higher accuracy than the traditional method of artificial inference [22].

The primary objective of this study is to assess the accuracy of using ML to predict whether Chinese adolescents have reached the age of criminal responsibility (16 years). The ML models incorporate the PL_2M_, Demijirjian_3M_, and I_3M_ indices. The models employed in this study include random forest (RF), decision tree (DT), support vector machine (SVM), K-nearest neighbours (KNN), Bernoulli naive Bayes (BNB), and logistic regression (LR).

## Materials and methods

### Samples

The measurements and evaluations of the three indices (PL_2M_, Demirjian_3M_, and I_3M_) were conducted following appropriate operational guidelines and regulations. The research was authorized by the Independent Ethics Committee of the Shanghai Ninth Hospital affiliated with Shanghai Jiao Tong University, School of Medicine (SH9H-2019-T75-3). The imaging data utilized in this study were obtained from previously treated outpatients at our institution. As such, the biological information and data have been deidentified and cannot be traced back to individual subjects, rendering it impossible to obtain informed consent from the participants. Therefore, this study was granted an exemption from obtaining informed consent by the Independent Ethics Committee of the Shanghai Ninth Hospital affiliated with Shanghai Jiao Tong University, School of Medicine.

This is a retrospective study of digital orthopantomographs (OPGs). OPGs were collected randomly from 665 children aged 12 to 20 in eastern China. The upper limit of 20 years was set because the development of the second and third molars in the lower jaw, which makes it clear to determine if someone is over 16, is mostly completed by that age. A total of 340 males and 325 females were divided into 9 groups by chronological age. OPGs were grouped by age and sex (Table 2).

**Table 2:** Age groups and sex distribution

Age Group	Female	Male	Total
12.00–12.99	35	37	72
13.00–13.99	43	38	81
14.00–14.99	41	40	81
15.00–15.99	37	40	77
16.00–16.99	37	38	75
17.00–17.99	29	33	62
18.00–18.99	34	47	81
19.00–19.99	36	42	78
20.00–20.99	33	25	58
Total	325	340	665

OPGs were taken using a KODAK 8000C Panoramic and Cephalometric Digital Dental X-ray Machine. Notably, all OPGs included contained both mandibular second and third molars. OPGs lacking these teeth were excluded since they are required for dental age estimation. Those who had undergone endodontic treatment for the mandibular third or second molar, had poor-quality OPGs, or had related diseases affecting jaw development were also excluded.

### Methods

First, all OPGs were anonymized and numbered with Arabic numerals before measurement and analysis.

**Fig. 1:**
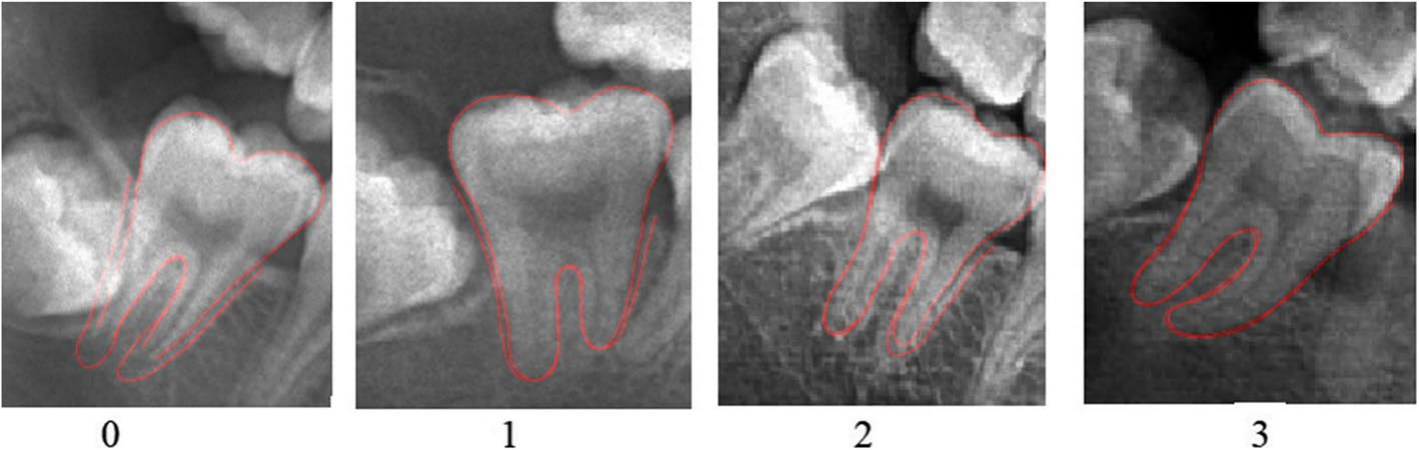
Pictures of the stages of radiographic visibility of the periodontal ligament in third molars

Y. Guo classified the periodontal ligament into the following 4 stages according to the visibility of the periodontal ligament of the outer parts of the second molar (PL_2M_) roots (mesial part of the mesial root and distal part of the distal root) (Fig. 1) [18]:

- Stage 0: The periodontal ligament is clearly visible around all tooth roots.
- Stage 1: The periodontal ligament on one root is indistinct or invisible from the tooth apex to more than half of the tooth root.
- Stage 2: The periodontal ligament of the entire tooth root is not visible, or the periodontal ligament around both tooth roots is partially invisible.
- Stage 3: The periodontal ligament on both tooth roots is barely visible.

In this study, Y. Guo's method was applied to second molars.

According to the Cameriere method [23], we divided the distance between the inside of the apex of the open root apex (A) by the length of the tooth (L) to calculate the dental maturity of the mandibular third molar, I_3M_. If the third molar is completely developed and the root apices are closed, I_3M_ = 0; otherwise, I_3M_ = A/L.

In addition, the Demirjian classification was applied to the third molar to determine whether individuals had reached 16 years of age (Demirjian_3M_). The degree of mineralization of the mandibular third molar was evaluated from stages A to H according to Demirjian classification [24].

Two trained observers independently evaluated OPGs blinded to biological and identity information except for the code of the subject. Two observers evaluated 20 random OPGs using the Y. Guo, Cameriere and Demirjian methods. The OPGs were reassessed by the same authors two months after the initial assessment. For I_3M_, the intraclass correlation coefficient (ICC) for the intrarater agreement was 0.926. Meanwhile, the ICC for the interrater agreement was 0.865. For PL_2M_ and Demirjian_3M_, the intrarater Kappa was 0.895, and the interrater Kappa was 0.828.

The following variables were incorporated to train six ML models: sex (s), PL_2M,_ I_3M_ and Demirjian_3M_. This was a binary classification problem focused on whether the subject was over 16 years of age, and the classification criterion was chronological age. Out-of-sample performance was assessed using the well-known K-fold cross-validation method. In this study, k was taken as 5. A 20% validation dataset was used during hyperparameter optimization to prevent overfitting. Hyperparameter tuning to obtain the best model was achieved by exploring multiple combinations using the GridSearchCV function. The hyperparameters described in Supplementary Table S1 were tuned.

### Statistical analysis

The sensitivity, specificity, overall accuracy and posttest probability were calculated to compare the accuracy of the abovementioned methods.

The date of birth was subtracted from the date the OPGs were obtained to calculate the chronological age. In this study, we used the estimated age as a dichotomous response variable (E = 0 if the person was less than 16 years old; otherwise, E = 1), and the predictors were sex (s), PL_2M,_ I_3M_ and Demirjian_3M_. Generalized linearity was derived from a logistic model to predict whether the person is under 16 years of age (E = 0) or not (E = 1).

The model's predictive accuracy was evaluated by the characteristic receiver operating characteristic (ROC) curve. All-important variables were applied to determine whether the individual was under 16. The area under the ROC curve (AUC) is a commonly used metric for evaluating the performance of binary classification models. The AUC ranges between 0.5 and 1, with values closer to 1 indicating better classifier performance, while a value of 0.5 indicates performance equivalent to random guessing. The AUC represents the probability that the classifier will correctly rank a randomly chosen positive example higher than a random negative one. Therefore, a higher AUC indicates better classifier performance.

Using the Bayesian theorem enables researchers to integrate regional characteristics and environmental factors, resulting in more accurate estimations that align with the specific conditions in China. Additionally, sensitivity and specificity tests comprehensively evaluate the model's performance, ensuring the accuracy of event classification across different age groups.

Tests were performed to derive sensitivity (${S}_{e}$, proportion of verified T = 1 events in teens 16 years and older) and specificity (${S}_{p}$, proportion of verified T = 0 events in teens younger than 16 years). The formula for the posttest probability according to Bayes theorem is as follows:

1 $$\mathrm{p}=\frac{{S}_{e}{p}_{0}}{{S}_{e}{\times p}_{0}+\left(1-{S}_{p}\right)\left(1-{p}_{0}\right)}$$

where p represents the posttest probability, and p_0_ corresponds to the prior probability or the initial probability, which refers to the proportion of the 12- to 20-year-olds to be over 16. This proportion of p_0_ was considered to be 0.630 overall. The value of p_0_ is calculated according to demographic data from the Nation Bureau of Statistics of the People's Republic of China (http://www.stats.gov.cn/tjsj/pcsj/). Data analysis and related icon production were performed using SPSS 25.0 (IBM Corp. Released 2017. IBM SPSS Statistics for Windows, Version 25.0. Armonk, NY: IBM Corp.), Pycharm 2021 and Python 3.8.2. The significance level was set at 5%.

## Results

**Fig. 2:**
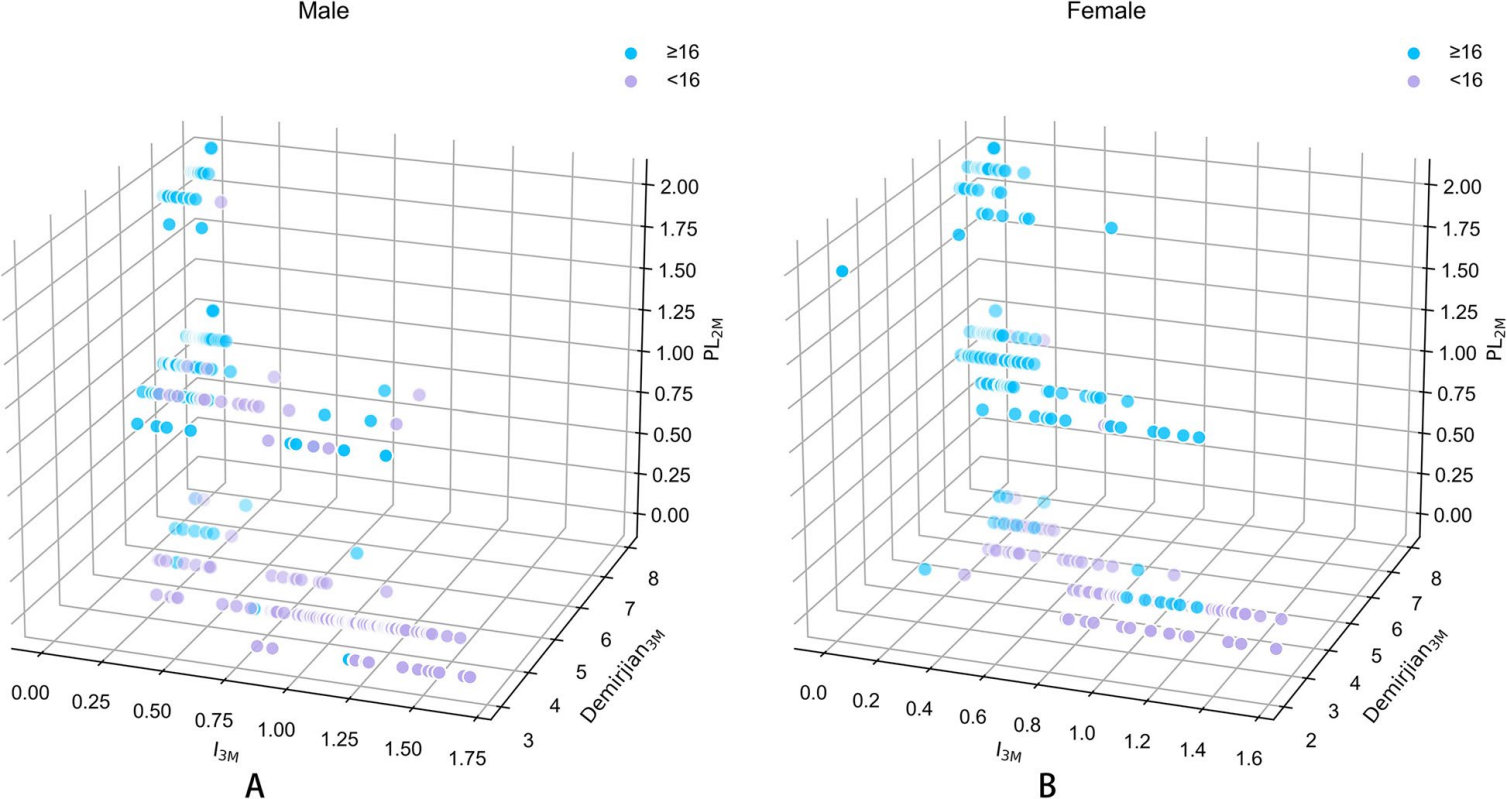
3D Scatter-plot of the relationship between age 16 and I_3M_, Demirjian_3M_ and PL_2M_ for (**A**) Male and (**B**) Female

The scatterplots in Fig. 2A and B show a clear trend in the distribution of the three dental developmental markers in relation to age for females and males, respectively. As chronological age increases, there is a general increase in the PL_2M_ and Demirjian_3M_ values and a general decrease in the I_3M_ values, indicating dental maturation. This trend was observed for both sexes, suggesting a potential association between these markers and age-related dental development.

**Fig. 3:**
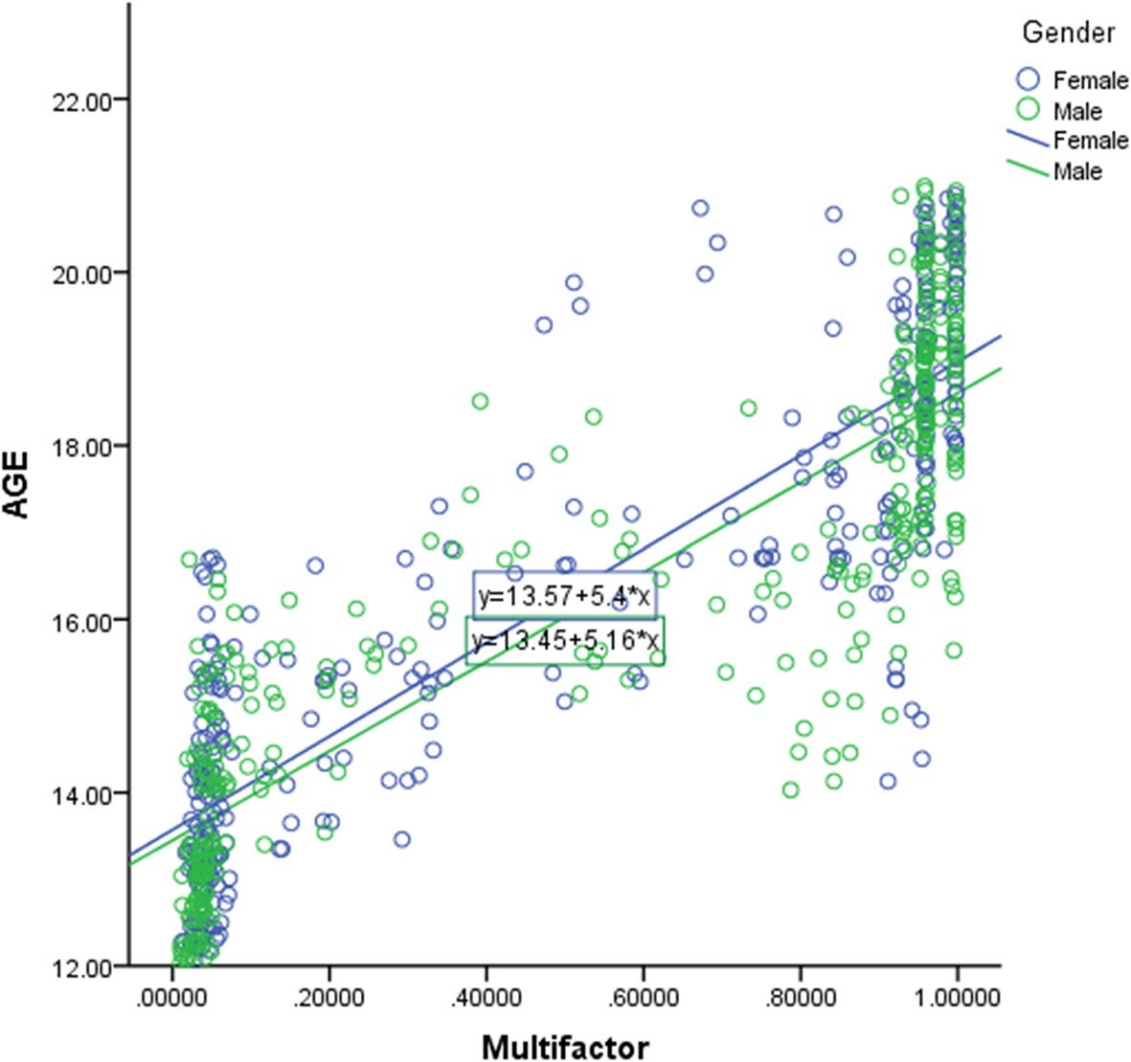
Scatter-plot of the relationship between age and multifactor

We calculated the Pearson correlation coefficient to quantify the relationship between sex and chronological age in relation to multiple factors (I_3M_, PL_2M_, and Demirjian_3M_). As shown in Fig. 3, the correlation coefficient was 0.749 (*p* < 0.001) for both males and females. This finding indicates a strong positive correlation between multiple factors and chronological age for both sexes. The statistically significant correlation supports the notion that these dental developmental markers are influenced by chronological age.

Notably, while these markers exhibit a clear linear trend with age, variability exists within each age group. Overall, the trend observed in the scatter plot supports using dental developmental markers for age estimation in forensic and clinical settings to determine whether an individual has reached the age of criminal responsibility (16 years).

The logistic regression model revealed that I_3M_, PL_2M_, and Demirjian_3M_ were significant predictors (*p* < 0.05); thus, they were included in the analysis. Conversely, sex was found to be nonsignificant (*p* = 0.163) and demonstrated no association with the determination of age over 16 from dental assessment (Table 3).

**Table 3:** Parameter estimates ofI_3M_, Demirjian_3M_, PL_2M_ and sex (s) as explanatory variables and ≥ 16 years (E = 1) and < 16 years (E = 0) age as dichotomous dependent variable on Logistic Regression

	B	Std. error	Wald statistics	df	P
I_3M_	-1.663	.627	7.043	1	.008
Demirjian_3M_	.516	.210	6.064	1	.014
PL_2M_	3.104	.303	105.071	1	.000
Sex	-.420	.301	1.947	1	.163
Constant	-3.205	1.378	5.408	1	.020

**Fig. 4:**
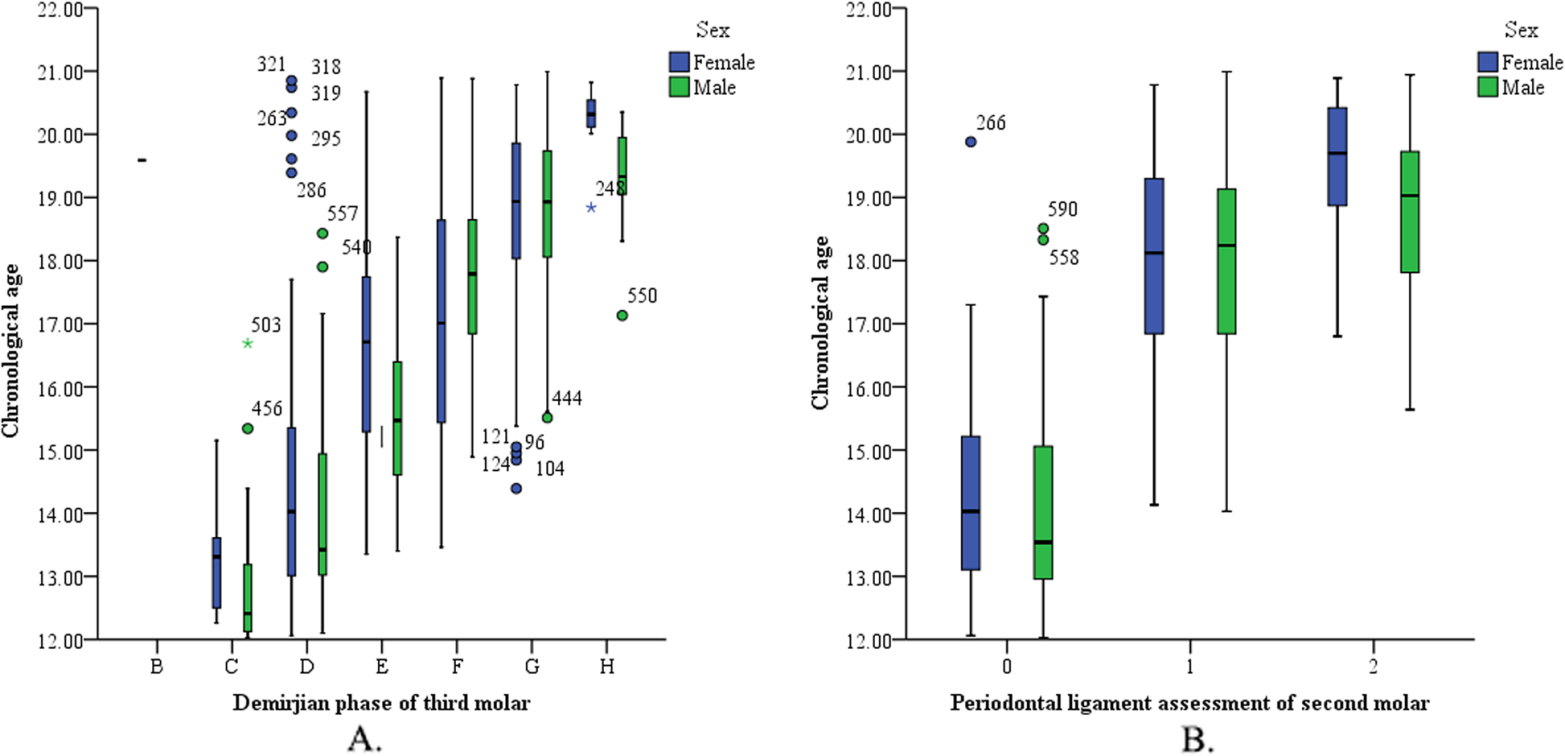
Boxplot of the relationship between age (years) and (**A**) Demirjian_3M_ and (**B**) PL_2M_

The cutoff of I_3M_ in this study was 0.33, which can be calculated by logistic regression (see Supplementary Table S2). Figure 4A and B demonstrate that chronological age increased as PL_2M_ and Demirjian_3M_ increased.

**Fig. 5:**
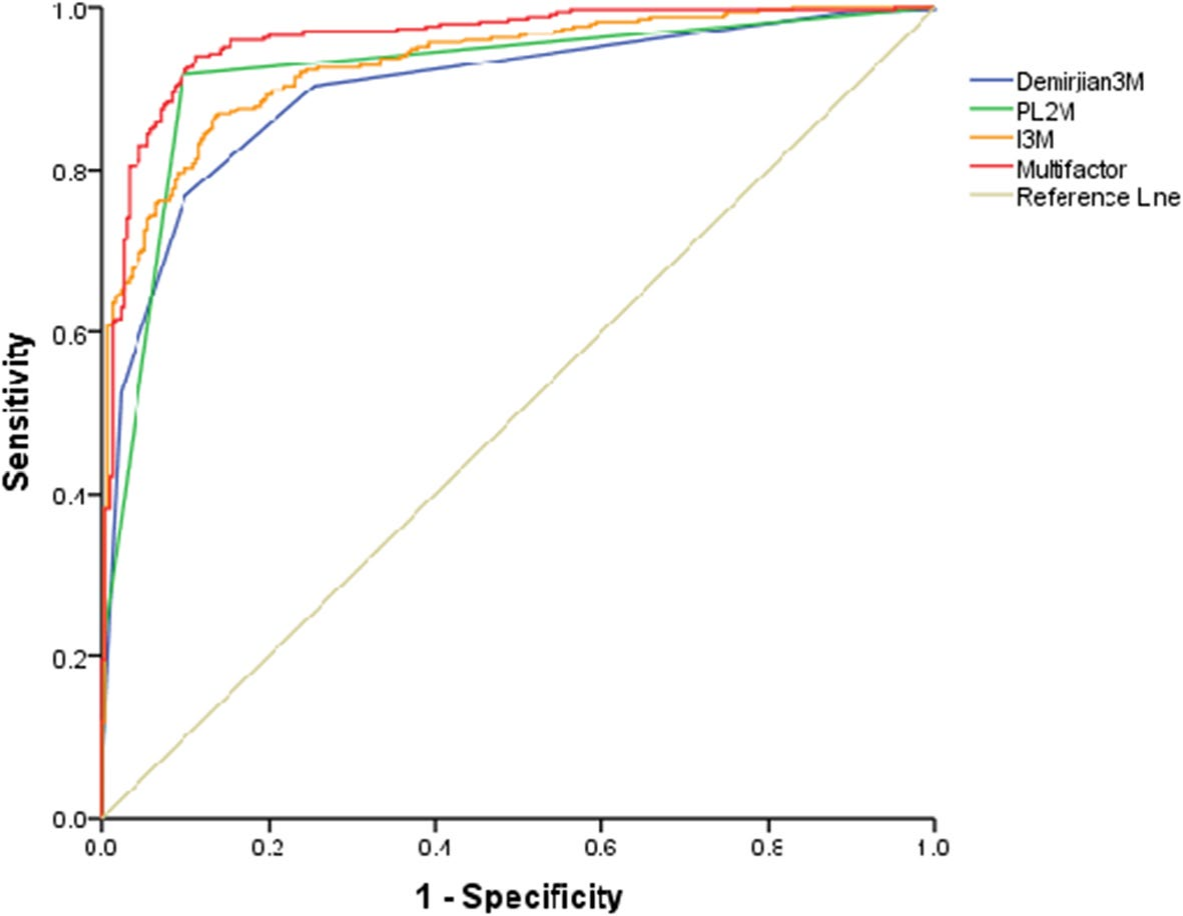
The receiver operating characteristic curve for indicating whether the individual is over 16 years by I_3M_, Demirjian_3M_, PL_2M_ and multifactor

Figure 5 displays the receiver operating characteristic (ROC) curves for I_3M_, PL_2M_, Demirjian_3M_, and the multiple factors. As shown in Fig. 3, the multifactor variable, which combines I_3M_, PL_2M_, and Demirjian_3M_, has the highest AUC, indicating superior performance in determining whether an individual is over 16. We performed a ROC analysis on the entire sample. Multifactor has the highest AUC, indicating that it has the best performance for 16-year-old discrimination.

Furthermore, we utilized six ML models in combination with I_3M_, PL_2M_, Demirjian_3M_, and sex to determine whether an individual is over 16.

**Fig. 6:**
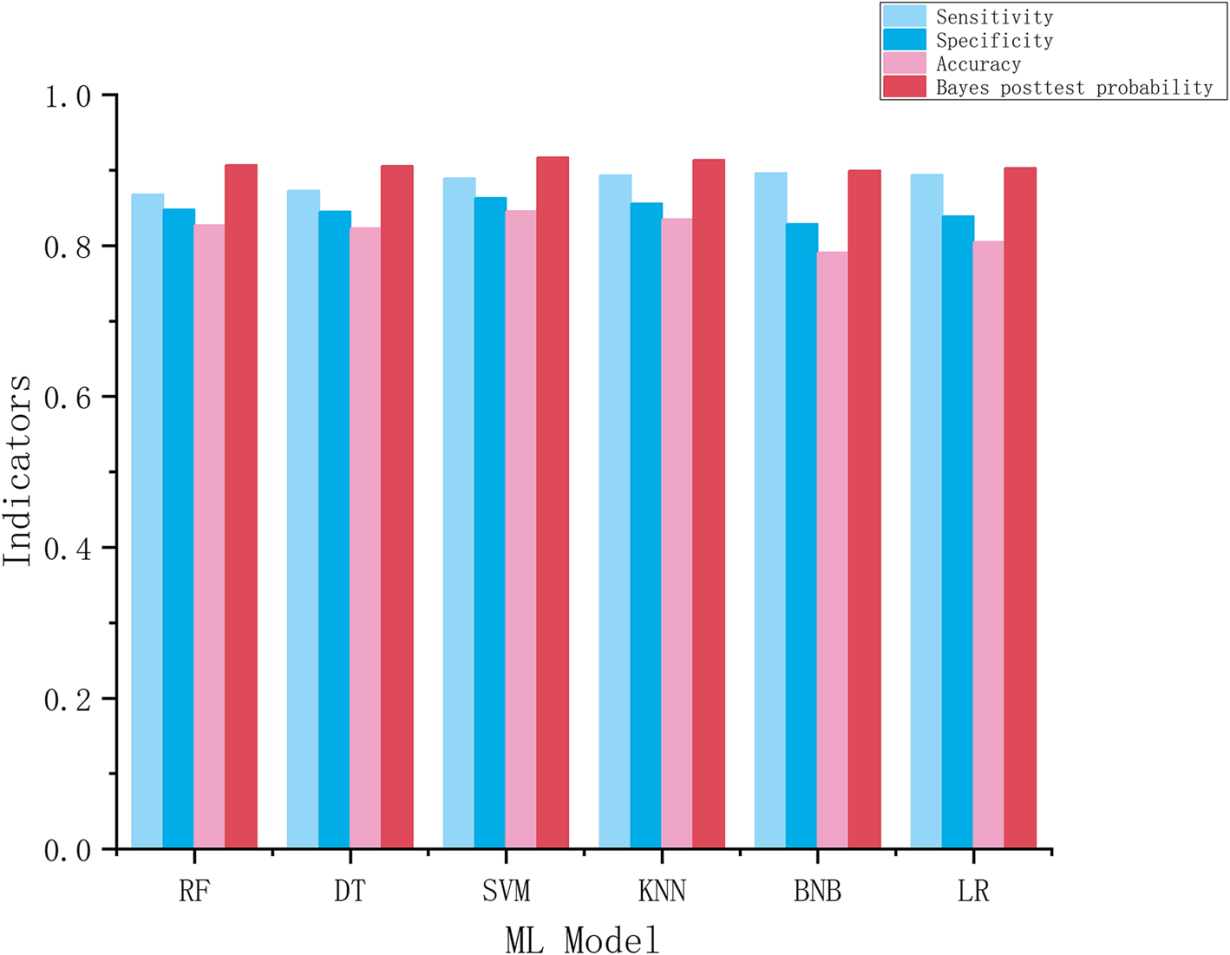
Sensitivity, specificity, accuracy and Bayes posttest probability of six ML models

Table 4 and Fig. 6 show the results of different the ML models for the binary classification problem of judging whether an individual is 16 or older. The results show that all models perform relatively well, with accuracies ranging from 0.791 to 0.846. However, there are some differences in performance when considering sensitivity, specificity, and Bayes posttest probability.

**Table 4:** The quantities to test the age of majority for six ML models

	RF	DT	SVM	KNN	BNB	LR
Sensitivity	0.868	0.873	0.889	0.893	0.896	0.894
Specificity	0.848	0.845	0.864	0.856	0.829	0.839
Accuracy	0.821	0.823	0.846	0.835	0.791	0.805
Bayes posttest probability	0.907	0.906	0.918	0.913	0.899	0.903

The models with the highest sensitivity were KNN and BNB, with values of 0.893 and 0.896, respectively. SVM had the highest specificity, with a value of 0.864, followed closely by KNN, with a value of 0.856. When considering the Bayes posttest probability, SVM had the highest value of 0.918, followed by KNN, with a value of 0.913. The lowest value for Bayes posttest probability was observed in BNB, with a value of 0.899.

Regarding overall performance, SVM had the highest accuracy (0.846), while BNB had the lowest (0.791). However, accuracy alone may be insufficient to evaluate the performance of a model. Sensitivity and specificity provide a better understanding of how well the model detects true positives and negatives.

In summary, the results suggest that SVM is the best model in terms of overall performance, having the highest accuracy and Bayes posttest probability and the second-highest values for sensitivity and specificity. BNB had the lowest performance overall, with the lowest accuracy and Bayes posttest probability and the second-lowest values for sensitivity and specificity.

## Discussion

Determining the age of adolescents holds great legal and medical significance, particularly considering that 16 years of age is associated with criminal responsibility in many countries and regions. Previous studies by Cameriere have suggested an I_3M_ threshold of 0.36 for determining whether an individual is 16 [16]. If I_3M_ ≤ 0.36, the person can be considered to have reached the age of 16, while values higher than 0.36 indicate that the person is below 16. However, multiple research reports have highlighted the need for recalibrating the I_3M_ threshold for each specific population [25, 26].

Therefore, in this study, we opted to rely solely on the I_3M_ measurement without using a fixed threshold and instead embraced ML techniques. We harnessed the advantage of automatic pattern recognition and correlation learning within the data by employing ML models, eliminating the need for manual threshold setting. This approach allowed the model to adapt more effectively to diverse datasets and populations, enhancing its versatility and adaptability. Furthermore, using ML methods enabled us to consider multiple features or indicators simultaneously, extending beyond the constraints of a single metric. For instance, our examination of three different data points (PL_2M_, Demirjian_3M_, and I_3M_) related to the second and third molars in the mandible provided a comprehensive approach, yielding more robust information and improving the accuracy and reliability of age determination.

Two types of errors can occur in classification tests: Type I errors, or false-negatives (FNs), and Type II errors, or false-positives (FPs). In our research, minimizing Type I and Type II errors proved crucial in safeguarding the rights of adolescents and ensuring accurate age determination [27]. The Youden index, which combines sensitivity and specificity, played a vital role in evaluating the overall performance of our binary classification models.

According to the Youden formula, the Youden index for Cameriere's suggested I_3M_ = 0.36 is 0.6 [16]. The SVM model displayed the highest Youden index value of 0.753 among our evaluated models. By incorporating ML, we observed an improvement in age determination performance compared to that of manual approaches.

Moreover, the SVM model consistently emerged as the most effective classifier based on the Youden index, aligning perfectly with the results obtained from the Bayes posttest probability analysis (0.917). This finding further strengthens the notion that the SVM model is likely the most suitable for determining whether an individual has reached the age of 16.

The performance of ML models in binary classification can vary due to factors such as model complexity and hyperparameter settings. SVM and KNN are recognized for their ability to handle complex patterns effectively, while DT and RF may have certain limitations in this regard, potentially explaining why the SVM model is suitable for the purpose of this study.

## Conclusion

The SVM model exhibited strong performance, as indicated by its high Youden index and Bayes posttest probability values. This model demonstrates a good trade-off between sensitivity and specificity and is well suited for age determination, specifically in determining whether an individual has reached the age of 16 years.

### Supplementary Information

**Additional file 1:** **Supplementary Table S1.** List of the tuned hyperparameters for each Machine Learning algorithm. For each hyperparameter, the values inside square brackets were explored by Grid Search. **Supplementary Table S2.** Parameter estimates for logistic model for I_3M._

## Data Availability

The datasets used and/or analyzed during the current study available from the corresponding author on reasonable request.
