# Supplementary material for: Using machine learning to determine age over 16 based on development of third molar and periodontal ligament of second molar
Source: BMC Oral Health. 2023 Sep 20;23:680. doi: 10.1186/s12903-023-03284-5 (PMC10510268; doi:10.1186/s12903-023-03284-5)
Supplement: Supplementary file 1 — Additional file 1: Supplementary Table S1. List of the tuned hyperparameters for each Machine Learning algorithm. For each hyperparameter, the values inside square brackets were explored by Grid Search. Supplementary Table S2. Parameter estimates for logistic model for I3M. [file 12903_2023_3284_MOESM1_ESM.docx]

Supplementary Table S1 List of the tuned hyperparameters for each Machine Learning algorithm. For each hyperparameter, the values inside square brackets were explored by Grid Search.

| K-Nearest Neighbors (KNN) | *n_neighbors*: the number of neighbors or K to use [12 to 20] *weights*: the weight function used in prediction [uniform or distance]  *algorithm*: type of algorithm used to compute the nearest neighbors [ball tree, kd tree or brute] |
| --- | --- |
| Decision Tree (DT) | *criterion*: the function to measure the quality of a split in the tree [mse, Friedman mse or mae]  *max_depth*: the maximum depth of the tree [6 to 8]  *max_features*: the number of features to consider when looking for the best split at a node [auto, sqrt, log2]  *min_samples_split*: the minimum number of samples required to split an internal node [2 to 5]  *min_samples_leaf:* the minimum number of samples required to be at a leaf node [2 to 5]  *splitter*: the strategy used to choose the split at each node [best or random] |
| Bernoulli Naive Bayes (BNB) | *alpha_1*: shape parameter for the Gamma distribution prior over the alpha parameter [1e^-15^, 1e^-10^, 1e^-8^, 1e^-4^, 1e^-3^, 1e^-2^, 1, 5, 10, 20] *alpha_2*: inverse scale parameter (rate parameter) for the Gamma distribution prior over the alpha parameter [1e^-15^, 1e^-10^, 1e^-8^, 1e^-4^, 1e^-3^, 1e^-2^, 1, 5, 10, 20] *lambda_1*: shape parameter for the Gamma distribution prior over the lambda parameter [1e^-15^, 1e^-10^, 1e^-8^, 1e^-4^, 1e^-3^, 1e^-2^, 1, 5, 10, 20] *lambda_2*: inverse scale parameter (rate parameter) for the Gamma distribution prior over the lambda parameter [1e^-15^, 1e^-10^, 1e^-8^, 1e^-4^, 1e^-3^, 1e^-2^, 1, 5, 10, 20] |
| random forests (RF) | n_estimators: the number of trees in the forest [5, 10, 15, 20, 30, 40, 50],  max_depth: the maximum depth of a tree [6 to 8]  max_features: the number of features to consider when looking for the best split at a node [auto, sqrt, log2]  min_samples_split: the minimum number of samples required to split an internal node [2 to 5]  min_samples_leaf: the minimum number of samples required to be at a leaf node [2 to 5]  bootstrap: whether bootstrap samples are used when building trees [True, False] |
| Support Vector Machine (SVM) | kernel: the kernel type used in the algorithm [polynomial, linear, rbf]  gamma: kernel coefficient [0.0001, 0.0005, 0.001, 0.01, 0.05, 0.1]  C: the regularization parameter. The higher the parameter the lower the regularization strength [1 to 10] |
| Logisitic Regression(LR) | penalty: the choice of regularization term [l1,l2]  solver: the solver type used in the algorithm [liblinear, newton-cg, lbfgs] |

Supplementary Table S2 Parameter estimates for logistic model for I_3M_

| Parameter | Value | Std.Error | df | p |
| --- | --- | --- | --- | --- |
| I_3M_ | -4.891 | 0.328 | 1 | <0.001 |
| Constant | 2.622 | 0.189 | 1 | <0.001 |
